# Draft genome sequencing of multidrug-resistant *Pseudomonas asiatica* strains isolated from dairy cows with clinical mastitis and their farm environment

**DOI:** 10.1128/mra.00907-24

**Published:** 2024-10-15

**Authors:** Taniya Sultana, Naim Siddique, Monira Rahaman, Md. Morshedur Rahman, ANM Aminoor Rahman, Anup Kumar Talukder, Ziban Chandra Das, M. Nazmul Hoque

**Affiliations:** 1 Molecular Biology and Bioinformatics Laboratory, Department of Gynecology, Obstetrics and Reproductive Health, Bangabandhu Sheikh Mujibur Rahman Agricultural University (BSMRAU), Gazipur, Bangladesh; The University of Arizona, Tucson, Arizona, USA

**Keywords:** draft genomes, multidrug resistant, *Pseudomonas asiatica*, clinical mastitis, dairy cows

## Abstract

We report, for the first time, the draft genomes of four multidrug-resistant *Pseudomonas asiatica* strains isolated from milk (2M1), feces (2F1 and 2F2), and farm soil (2S1) samples of dairy cows with clinical mastitis. The assembled genomes of these strains were 5.7 Mbp, 62.8% G + C content, and 50× genome coverage.

## ANNOUNCEMENT

Mastitis in cows is one of the most widespread and economically impactful diseases in the dairy industry (1). A wide range of etiological agents are accountable for causing mastitis (2). *Pseudomonas* spp. is considered as one of the environmental mastitis-causing pathogens (3). *P. asiatica*, a newly identified species within *P. putida* group, and closely related to *P. putida* and *P. monteilii*, has been isolated from hospitalized patients in Japan and Myanmar (4, 5). This study provides the first documented evidence of *P. asiatica* associated with clinical mastitis in dairy cows, marking a significant and novel discovery with potential implications for dairy herd management and disease control.

Milk, feces, and soil samples were collected from a cow with clinical mastitis (CM), diagnosed *via* the California Mastitis Test (CMT) (6), at a dairy farm in Gazipur, Bangladesh (24.09° N, 90.41° E). A total of 110 samples were collected, consisting of 45 from CM milk, 35 from feces, and 30 from farm soil, representing both small- and large-scale dairy operations. Milk samples were collected in accordance with the 2014 standards set by the National Mastitis Council (7). Freshly deposited wet fecal materials were gathered directly from the ground. During morning milking (8:00–10:00 a.m.), approximately 10 mL of milk and 5 g of feces and soil were aseptically collected from each cow in sterile Falcon tubes, with meticulous attention to pre-sampling disinfection of teat ends and rigorous adherence to hygiene protocols. To isolate and phenotypically identify *P. asiatica*, samples were first enriched in nutrient broth (Oxoid, UK) incubating overnight at 37°C. The bacteria-enriched broth was then serially diluted up to 10^−3^ fold and plated on nutrient agar (Oxoid, UK), followed by overnight incubation at 37°C. Pure colonies were obtained by streaking on selective tryptic soy agar (TSA) (Oxoid, UK) plates by incubating at 37°C for 24 h (8). Phenotypic identification of the isolates was performed by examining colony morphology and Gram staining (Gram-negative, rod shape, pink colony) (9). The species-level identification of *P. asiatica* strains 2M1, 2F1, 2F2, and 2S1 was carried out using the VITEK-2 system (v9.01) (10). These isolates exhibited multidrug resistance (resistance to >3 antibiotic classes), and the highest resistance was found against imipenem, ampicillin, oxacillin, sulfonamides, nitrofurantoin, azithromycin, tetracycline, and cefoxitin following the Clinical Laboratory Standards Institute M100 2023 (CLSI M100 2023) guidelines (11). A pure colony from each multidrug-resistant *P. asiatica* isolate was grown in nutrient broth overnight at 37°C. DNA was extracted using the QIAamp DNA Mini Kit (QIAGEN, Germany) (12). Whole-genome sequencing libraries were then prepared from 1 ng of extracted DNA per isolate using the Nextera DNA Flex Kit (Illumina, USA). The libraries were sequenced on the Illumina MiSeq platform using a 2 × 250 bp paired-end read configuration. Raw reads were subjected to trimming using Trimmomatic v0.39 (13) and underwent quality assessment with FastQC v0.11.7 (14). The genomes were assembled with SPAdes v3.15.5 (15) and subsequently annotated using the NCBI Prokaryotic Genome Annotation Pipeline v6.6 (16). Genome completeness, virulence factor genes (VFGs), antimicrobial resistance genes (ARGs), and metabolic functions in the draft genomes were predicted by using CheckM v1.2.2 (17), VFDB v6.0 (18), ResFinder 4.0 (19), and RAST server (20), respectively. Default parameters were used in all analyses unless specified otherwise.

Table 1 provides detailed information for genome assembly and annotation, including genome size, number of contigs, *N_50_* values, and the number of annotated genes, offering a comprehensive overview of the assembly quality and completeness. The draft genome size of these four multidrug-resistant *P. asiatica* strains is approximately 5.7 Mbp, each with a G + C content of 62.8% and 50× coverage. Each of these four genomes contains approximately 370 subsystems, 5,100 protein-coding genes, 30 VFGs, and 11 ARGs indicating resistance to multiple antibiotics (Table 1).

**TABLE 1:** Genomic features of the *P. asiatica* strains isolated from milk, feces, and farm soil of dairy cows with clinical mastitis

Genomic features	*P. asiatica* strains			
	2 M1	2 F1	2 F2	2 S1
No. of reads	614,714	2,150,466	1.821,970	2,423,428
Genome size (bp)	5,672,760	5,663,297	5,660,490	5,665,922
Genome coverage (×)	50	50	50	50
G + C content (%)	62.8	62.8	62.8	62.8
Number of contigs	80	75	75	80
Genome completeness (%)	100 (0.11% contamination)	100 (0.11% contamination)	100 (0.11% contamination)	100 (0.11% contamination)
Contig *N*_50_ (bp)	122,302	150,834	150,834	150.767
Coding sequences (CDSs)	5,188	5,170	5,174	5,173
Protein-coding genes	5,132	5,116	5,120	5,119
No. of VFGs	30	30	30	30
No. of ARGs	11	11	11	11
Genome accessions	JAXUBP000000000	JAXUBN000000000	JAXUBM000000000	JAXUBO000000000
SRA accessions	SRR26993013	SRR26993011	SRR26993010	SRR26993012

## Data Availability

The whole-genome shotgun project of the *Pseudomonas asiatica* strains has been deposited in GenBank and the NCBI Sequence Read Archive (SRA) under BioProject accession number PRJNA1046646. The SRA accessions are listed in Table 1. The version described in this paper are versions JAXUBP000000000.1, JAXUBN000000000.1, JAXUBM000000000.1, and JAXUBO000000000.1.
